# Synthesis and preclinical evaluation of novel ^99m^Tc-labeled PSMA ligands for radioguided surgery of prostate cancer

**DOI:** 10.1186/s13550-022-00942-7

**Published:** 2023-01-16

**Authors:** Jan-Philip Kunert, Max Müller, Thomas Günther, León Stopper, Nicole Urtz-Urban, Roswitha Beck, Hans-Jürgen Wester

**Affiliations:** Chair of Pharmaceutical Radiochemistry, Department of Chemistry, Technical University of Munich (TUM), Walther-Meißner-Str 3, 85748 Garching, Germany

**Keywords:** Technetium-99m, PSMA, Radioguided surgery, Prostate cancer

## Abstract

**Background:**

Radioguided surgery (RGS) has recently emerged as a valuable new tool in the management of recurrent prostate cancer (PCa). After preoperative injection of a ^99m^Tc-labeled prostate-specific membrane antigen (PSMA) inhibitor, radioguided intraoperative identification and resection of lesions is facilitated by means of suitable γ-probes. First clinical experiences show the feasibility of RGS and suggest superiority over conventional lymph node dissection in recurrent PCa. However, commonly used [^99m^Tc]Tc-PSMA-I&S exhibits slow whole-body clearance, thus hampering optimal tumor-to-background ratios (TBR) during surgery. We therefore aimed to develop novel ^99m^Tc-labeled, PSMA-targeted radioligands with optimized pharmacokinetic profile to increase TBR at the time of surgery.

**Methods:**

Three ^99m^Tc-labeled N4-PSMA ligands were preclinically evaluated and compared to [^99m^Tc]Tc-PSMA-I&S. PSMA affinity (IC_50_) and internalization were determined on LNCaP cells. Lipophilicity was assessed by means of the distribution coefficient log*D*_7.4_ and an ultrafiltration method was used to determine binding to human plasma proteins. Biodistribution studies and static *µ*SPECT/CT-imaging were performed at 6 h p.i. on LNCaP tumor-bearing CB17-SCID mice.

**Results:**

The novel N4-PSMA tracers were readily labeled with [^99m^Tc]TcO_4_^−^ with RCP > 95%. Comparable and high PSMA affinity was observed for all [^99m^Tc]Tc-N4-PSMA-ligands. The ligands showed variable binding to human plasma and medium to low lipophilicity (log*D*_7.4_ − 2.6 to − 3.4), both consistently decreased compared to [^99m^Tc]Tc-PSMA-I&S. Biodistribution studies revealed comparable tumor uptake among all [^99m^Tc]Tc-N4-PSMA-ligands and [^99m^Tc]Tc-PSMA-I&S, while clearance from most organs was superior for the novel tracers. Accordingly, increased TBR were achieved. [^99m^Tc]Tc-N4-PSMA-12 showed higher TBR than [^99m^Tc]Tc-PSMA-I&S for blood and all evaluated tissue. In addition, a procedure suitable for routine clinical production of [^99m^Tc]Tc-N4-PSMA-12 was established. Labeling with 553 ± 187 MBq was achieved with RCP of 98.5 ± 0.6% (*n* = 10).

**Conclusion:**

High tumor accumulation and favorable clearance from blood and non-target tissue make [^99m^Tc]Tc-N4-PSMA-12 an attractive tracer for RGS, possibly superior to currently established [^99m^Tc]Tc-PSMA-I&S. Its GMP-production according to a method presented here and first clinical investigations with this novel radioligand is highly recommended.

**Supplementary Information:**

The online version contains supplementary material available at 10.1186/s13550-022-00942-7.

## Introduction

Throughout the last decade, radiopharmaceuticals targeting prostate-specific membrane antigen (PSMA) have become an integral part in clinical management of prostate cancer (PCa) [1, 2]. Almost in parallel with the clinical success of diagnostic tracers such as [^68^Ga]Ga-PSMA-11 [3] and [^18^F]DCFPyL [4] for positron emission tomography (PET) or [^99m^Tc]Tc-MIP-1404 [5] for single-photon emission computed tomography (SPECT) imaging, several therapeutic compounds entered the stage, among them ^177^Lu-labeled PSMA-I&T and PSMA-617 [6, 7]. In addition, several distinct therapeutic approaches have found their way into preclinical and clinical research, among them long-acting albumin-binding PSMA-ligands [8, 9], targeted alpha therapy [10] or so-called tandem therapy combining [^225^Ac]Ac- and [^177^Lu]Lu-PSMA-617 [11]. The recent approval of [^177^Lu]Lu-PSMA-617 (Pluvicto™, Novartis) for RLT of metastatic castration-resistant PCa (mCRPC) represents a milestone for nuclear medicine, broadens the armamentarium of oncologists, and might pave the way for further approved targeted therapeutic radiopharmaceuticals.

Radioguided surgery (RGS) is another therapeutic intervention successfully harnessing the potential of radioactive PSMA-targeted probes [12]. Patients with early biochemical recurrence after radical prostatectomy that show only regional pelvic lymph node metastases (LNM) in PSMA-PET imaging can benefit from radioguided salvage lymph node dissection (sLND) to delay disease progression and future systemic treatment [13]. In contrast to conventional sLND, a γ-emitting PSMA-targeted radioligand is intravenously injected up to 24 h prior to surgery. With the help of a γ-probe, localization and resection of metastatic lymph nodes are facilitated during surgery, which is especially useful in the case of small or atypically localized lesions. Furthermore, resected tissue can be identified instantly by ex vivo γ-probe measurements to confirm the successful removal of tumor-infested tissue. After initial proof-of-concept with [^111^In]In-PSMA-I&T [14], PSMA-RGS has been carried out with [^99m^Tc]Tc-PSMA-I&S [15], owing to its similar performance in vivo and more favorable radiation properties, the more common allowance to work with ^99m^Tc-tracers in surgery rooms, lower costs and higher availability of [^99m^Tc]TcO_4_^−^ compared to [^111^In]InCl_3_. A recent study with 121 patients by Horn et al. described the successful removal of preoperatively identified lesions in 99% of patients and a complete biochemical response in 66% of patients [16], which confirms results reported earlier by Maurer et al. for a smaller group of patients [13]. In the latter study, even additional lesions not previously detected on PSMA-PET could be removed, which highlights the potential of RGS for sLND [13]. However, prospective clinical trials such as the TRACE study (NCT03857113) are required to accurately assess the long-term outcome and added benefit of RGS for PCa patients.

Whether conventional or radioguided, the main limitation of sLND procedures is constituted in the incomplete resection of metastatic lesions. Even though state-of-the-art PSMA radio-guidance using [^99m^Tc]Tc-PSMA-I&S showed superior short-term efficacy in terms of biochemical response in a prospective study by Knipper et al. [17], the still insufficient sensitivity with regard to micro-metastatic lesions seems to be a major reason for recurrent disease [13, 18]. Technological developments like robot-assisted laparoscopic surgery implying drop-in or click-on γ-probes [19] and more differentiated criteria for patient selection [20] can certainly move the application forward. However, on a radiopharmaceutical level we identified a pending need of improvement, namely a radiotracer providing higher contrast and lower background signal than currently applied [^99m^Tc]Tc-PSMA-I&S. Its comparably slow whole-body clearance and partial hepatobiliary excretion is caused by high plasma protein binding (PPB) of 94% and reduced hydrophilicity compared to DOTA-based PSMA-radiochelated tracers [15]. As a result, unspecific background signal hampers both contrast in early SPECT-imaging and accurate detection during surgery [21, 22]. Thus, often [^99m^Tc]Tc-PSMA-I&S is not used to reliably identify LNMs in situ during surgery, but rather to confirm PSMA-positivity of resected tissue ex vivo. However, technically a more accurate lesion detection even in the surgical field should be feasible if TBR are sufficiently high, and thus, even smaller lesions might become detectable with an appropriately performing radiotracer. In spite of the known limitations of [^99m^Tc]Tc-PSMA-I&S, to our knowledge, no dedicated optimization of a ^99m^Tc-labeled PSMA-ligand intended for RGS application has been performed so far.

**Fig. 1:**
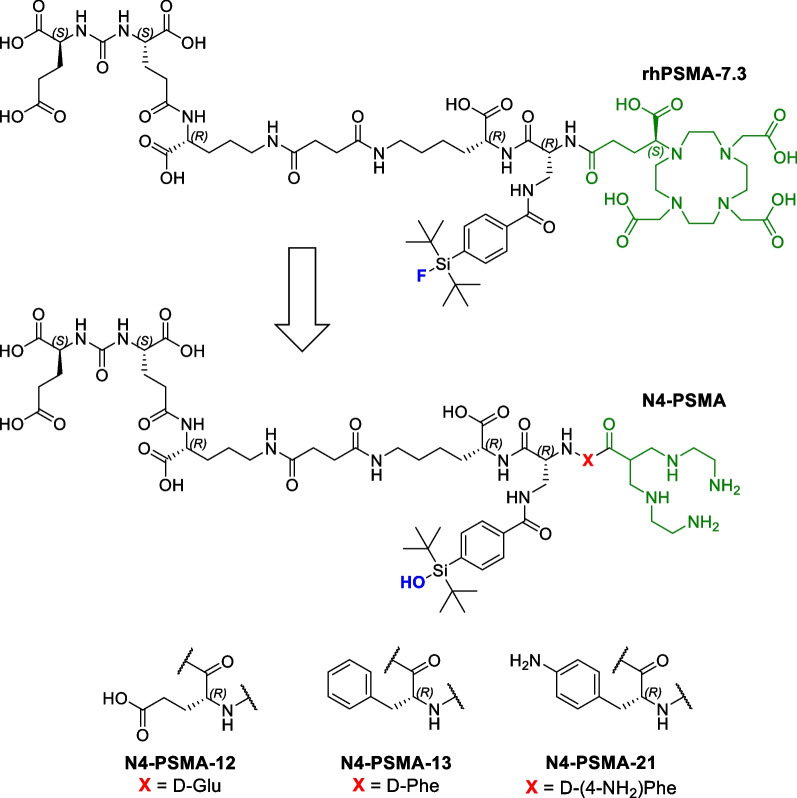
Molecular structures of the novel N4-PSMA ligands were derived from rhPSMA-7.3 by substitution of (*S*)-DOTAGA with a tetraamine (N4) chelator for complexation of technetium-99m (indicated in green) and incorporation of a less lipophilic, hydrolized derivative of the silicon fluoride acceptor-moiety (indicated in blue). A variable amino acid (X, red) is introduced in N4-PSMA-12, N4-PSMA-13 and N4-PSMA-21 to modulate their pharmacokinetic properties

To address this medical need, we developed a novel series of ^99m^Tc-labeled PSMA-targeted radioligands for RGS aiming for an improved pharmacokinetic behavior. The optimized ligand structure was conceptually designed and comprises a common PSMA-inhibitor motif derived from highly potent radiohybrid PSMA diagnostics [23] and therapeutics [24], a tetraamine (N4) chelator for reliable complexation of technetium-99m, and a variable amino acid for the modulation of pharmacokinetic properties of the peptide (Fig. 1). Such rational and iterative modifications constitute the core of radiopharmaceutical structure development and a multitude of studies have brought forth interesting PSMA radioligands for manifold applications using that approach [25–27]. As we aimed for differential (and primarily reduced) lipophilicity and plasma protein binding of the novel ligands, d-glutamate (d-Glu; negatively charged), d-phenylalanine (d-Phe; aromatic, uncharged), and 4-amino-d-phenylalanine (d-(4-NH_2_)Phe; aromatic, positively charged) were used as variable amino acids to introduce different charges and optional aromatic moieties.

In this study, we report the comparative preclinical evaluation of three ^99m^Tc-labeled N4-PSMA ligands in vitro and in vivo. All experiments were performed in comparison with [^99m^Tc]Tc-PSMA-I&S, the current clinical standard for PSMA-targeted RGS. In preparation of first clinical studies, we furthermore established a labeling procedure of the most promising derivative at patient scale that should be suited for routine clinical radioligand production.

## Materials and methods

Further general information and a detailed description of ligand synthesis are provided in Additional file 1.

### Radiolabeling

^99m^Tc-Labeling of N4-PSMA ligands was carried out by addition of 1 nmol peptide precursor (0.5 mM in DMSO) to a mixture of 0.05 M Na_2_HPO_4_ (12.5 µL, in Tracepur®-water, pH 9.25) and 0.1 M disodium citrate sesquihydrate (1.5 µL, in Tracepur®-water) in saline. After addition of a freshly prepared solution of SnCl_2_ (2.5 µL, 1 mg/mL in ethanol), [^99m^Tc]TcO_4_^−^ (40 MBq/nmol) in saline was added and the labeling solution (final volume 250 µL) was heated to 95 °C for 15 min. Subsequently, 10 µL of 1 M sodium ascorbate (in PBS) was added and quality control was performed using radio-TLC and radio-*RP*-HPLC (UV-detection at 220 nm). A slightly modified protocol for radiolabeling at patient scale and labeling of PSMA-I&S are described in Additional file 1.

### Lipophilicity and binding to human plasma

The lipophilicity of ^99m^Tc-labeled PSMA ligands, expressed as distribution coefficient (log*D*_7.4_), was determined using the shake-flask method. A solution of approximately 1 MBq of radioligand in 1 mL of a 1:1 mixture of PBS (pH = 7.4) and *n*-octanol (*n* = 8) was vortexed vigorously for 3 min. After centrifugation at 9000 rpm for 5 min aliquots of both phases (200 µL *n*-octanol, 50 µL PBS) were collected and the activity was quantified in a γ-counter. log*D*_7.4_ values were calculated as decadic logarithm of the activity concentration ratio between the *n*-octanol phase and the aqueous phase. Data are given as mean ± standard deviation (SD).

Binding to human plasma was determined by incubation of radioligands in human plasma (2 nM concentration, 37 °C for 30 min) and subsequent ultrafiltration (250 µL aliquots, 3200 rpm, 40 min) in Centrifree® ultrafiltration devices (Merck Millipore, Cork, Ireland). The fraction bound to human plasma proteins was calculated as the ratio of unfiltered activity and the total activity in the ultrafiltration device. Measurements were performed in three independent experiments, each with two replicates (*n* = 6, data are given as mean ± SD). All values were corrected for non-specific binding (control experiments in PBS).

### Determinations of affinities (IC_50_) and internalization studies

Competitive binding studies were carried out in analogy to a previously reported procedure [28]. As a modification the non-radiolabeled standard competitor (((*S*)-1-carboxy-5-(4-(iodo)benzamido)pentyl)carbamoyl)-l-glutamic acid (IBA-KuE) was applied in increasing concentrations (10^–5^–10^–11^ M/well, *n* = 3 each) whereas the novel PSMA-binding compounds of interest were applied as ^99m^Tc-labeled radioligands (0.2 nM/well). In this inversed experimental approach, higher values correspond to higher affinities and are referred to as inverse IC_50_. Poly-L-lysine coated 24-well plates were used (*n* = 3). Data are given as mean ± SD. To determine the cellular uptake of the ^99m^Tc-labeled PSMA ligands into LNCaP cells by means of PSMA-mediated internalization at 1 h, a previously reported protocol was applied with assay concentrations of 1.0 nM and 0.2 nM for ^99m^Tc-labeled PSMA ligands and the reference compound [^125^I]IBA-KuE, respectively [28]. Data are corrected for non-specific binding and normalized to the specific internalization of the reference. Results are given as mean ± SD.

### In vivo experiments

All animal experiments were conducted in accordance with general animal welfare regulations in Germany (German animal protection act, in the edition of the announcement, dated May 18th, 2006, as amended by Article 280 of June 19th 2020, approval no. ROB-55.2-1-2532.Vet_02-18-109 by the General Administration of Upper Bavaria) and the institutional guidelines for the care and use of animals. Male CB17-SCID mice were purchased from Charles River (Sulzfeld, Germany) and arrived at the in-house animal facility to acclimate at least 1 week before the start of the experiment. Tumor xenografts were established by subcutaneous inoculation of LNCaP cells (approx. 2 × 10^7^ cells in 200 µL of a 1:1 mixture of Cultrex BME (R&D Systems, Minneapolis, United States) and DMEM/Ham’s F-12) onto the right shoulder of 6–8 weeks old male CB17-SCID mice. The animals were used for experiments when tumors had grown to a size of 5–10 mm in diameter. Criteria for the exclusion of animals from the experiment were weight loss higher than 20%, tumor size above 1.5 cm^3^, ulceration of the tumor, respiratory distress or change of behavior. These criteria did not apply to any mouse. Neither randomisation nor blinding was applied in the allocation of the experiments. Quarterly health monitoring was performed according to the FELASA recommendations.

#### Biodistribution studies

The ^99m^Tc-labeled radioligands (2.7 ± 0.7 MBq, 82 ± 20 pmol) were injected into a lateral tail vein of LNCaP-tumor bearing mice (*n* = 4–5) under isoflurane anesthesia. Animals were sacrificed at 6 h post injection (p.i.) by carbon dioxide inhalation and blood withdrawal via cardiac puncture. Blood and tissues of interest were collected, weighed and the activity measured in a γ-counter. Radioligand uptake is given in percent of the injected dose per gram of tissue (% ID/g) and results are presented as mean ± SD. Further details on the analysis of biodistribution data are provided in Additional file 1: Fig. S1.

#### µSPECT/CT imaging

Static imaging of sacrificed animals was performed on a VECTor4 small-animal SPECT/PET/CT/OI scanner from MILabs (Utrecht, Netherlands) directly after blood collection with an acquisition time of 45 min using an HE-GP-RM collimator and a step-wise multiplanar bed movement via MILabs acquisition software (v11.00 and v12.26). Imaging data were reconstructed using MILabs-Reconstruction software (v12.00) and image analysis was performed with PMOD4.0 (PMOD technologies LLC, Zurich, Switzerland). Animals were subjected to biodistribution studies after imaging.

### Data analysis

Acquired data were statistically analyzed performing a one-way analysis of variances (ANOVA) followed by a Tukey’s multiple comparison post-test using OriginPro software (version 9.7) from OriginLab Corporation (Northampton, United States). Pairwise statistical comparisons were performed applying the two-sample student’s *t*-test in Microsoft Excel (Redmond, United States). Acquired *P* values of < 0.05 were considered statistically significant.

## Results

### Synthesis and radiolabeling

The novel PSMA ligands were synthesized using a mixed solution/solid phase synthetic approach and obtained with chemical purity of > 98% in yields of 29%, 25% and 21%, respectively. Compound identity was confirmed by mass spectrometry. Radiolabeling with [^99m^Tc]TcO_4_^−^ resulted in radiochemical purities (RCP) of > 95% as determined by radio-TLC and radio-*RP*-HPLC. Up-scaling of the reported labeling protocol for patient-scale production of [^99m^Tc]Tc-N4-PSMA-12 was found to be feasible, however proportionally reduced amounts of stannous chloride were applied to prevent the formation of colloidal technetium-species. Under consideration of requirements of a clinical workflow for routine radiosynthesis, labeling of N4-PSMA-12 (20 µg, 15 nmol) with activities of 194–810 MBq (553 ± 187 MBq, mean ± SD, *n* = 10) reproducibly yielded the desired radioligand with a RCP of 98.5 ± 0.6% (range, 97.6–99.2%, *n* = 10). Detailed information on single radiolabelings is provided in Additional file 1: Table S1.

### In Vitro Characterization

**Fig. 2:**
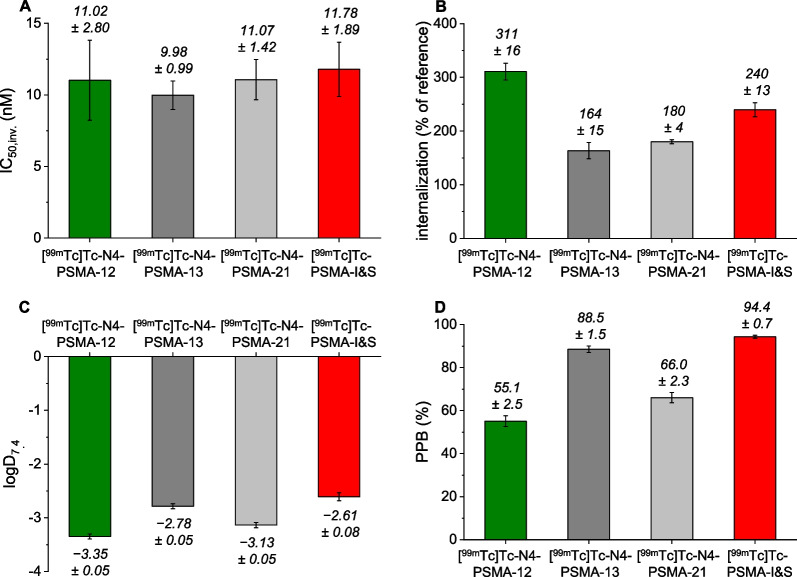
In vitro characterization of [^99m^Tc]Tc-N4-PSMA-12, [^99m^Tc]Tc-N4-PSMA-13, [^99m^Tc]Tc-N4-PSMA-21 and [^99m^Tc]Tc-PSMA-I&S: **A** Binding affinity to PSMA (IC_50,inv._ (nM), 1 h, 4 °C, *n* = 3); **B** PSMA-mediated internalization (1 h, 37 °C, (% of the reference [.^125^I]IBA-KuE), *n* = 3); **C** lipophilicity expressed as log*D*_7.4_ (*n*-octanol/PBS, pH 7.4, *n* = 8); **D** binding to human plasma (PPB) (incubation at 37 °C for 30 min, determination via ultrafiltration (%), *n* = 6)

Results of the in vitro characterization of [^99m^Tc]Tc-labeled N4-PSMA ligands and the reference [^99m^Tc]Tc-PSMA-I&S are summarized in Fig. 2 and Additional file 1: Table S2. PSMA affinity was assessed via determination of the inverse IC_50_ (IC_50,inv_). Irrespective of the variable amino acid, all radioligands showed high PSMA affinity displayed by low nanomolar IC_50,inv._ values (range, 10.0–11.8 nM) with no statistically significant difference (*P* > 0.66). In contrast, PSMA mediated internalization, expressed as percentage of the reference compound [^125^I]IBA-KuE, was significantly influenced by the variable amino acid. [^99m^Tc]Tc-N4-PSMA-12 (311 ± 16%) exhibited 1.3-fold higher internalization than [^99m^Tc]Tc-PSMA-I&S (240 ± 13%), and an even 1.9- and 1.7-fold higher value than [^99m^Tc]Tc-N4-PSMA-13 (164 ± 15%) and [^99m^Tc]Tc-N4-PSMA-21 (180 ± 4%), respectively. In comparison with [^99m^Tc]Tc-PSMA-I&S, all novel [^99m^Tc]Tc-N4-PSMA compounds showed increased hydrophilicity, expressed by the distribution coefficient (log*D*_*7.4*_). Within the group of N4-bearing radioligands, highest lipophilicity was observed for [^99m^Tc]Tc-N4-PSMA-13 (log*D*_7.4_ =  − 2.78 ± 0.05), comprising an aromatic d-Phe residue, followed by [^99m^Tc]Tc-N4-PSMA-21 (log*D*_7.4_ =  − 3.13 ± 0.05, d-(4-NH_2_)-Phe) and [^99m^Tc]Tc-N4-PSMA-12 (log*D*_7.4_ =  − 3.35 ± 0.05, d-Glu). Compared to [^99m^Tc]Tc-PSMA-I&S, binding to human plasma was significantly reduced for all [^99m^Tc]Tc-N4-PSMA tracers (94.4% vs 55.1–88.5%), and followed the same trend as described for lipophilicity. Thus, the highest hydrophilicity (log*D*_7.4_ =  − 3.35 ± 0.05) and lowest binding to human plasma proteins (55.1 ± 2.5%) was both found for [^99m^Tc]Tc-N4-PSMA-12 (*P* < 0.001 for both parameters).

### In vivo* characterization*

#### Biodistribution studies

**Fig. 3:**
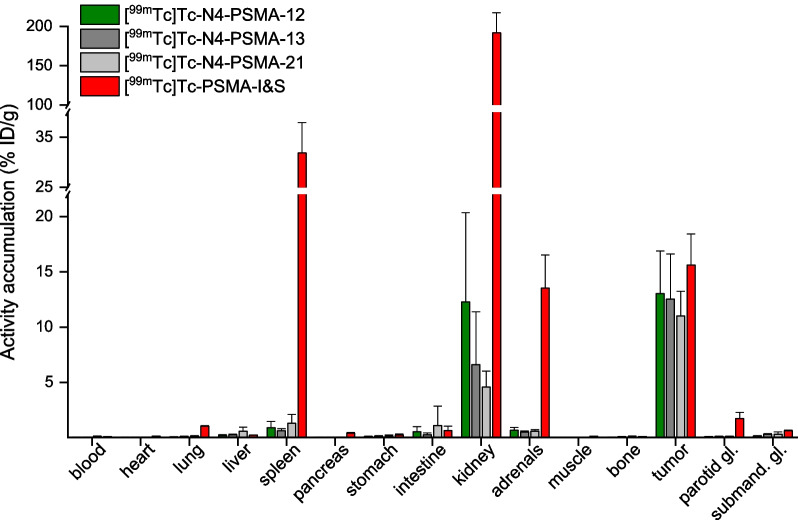
Ex vivo biodistribution data of ^99m^Tc-labeled N4-PSMA derivatives and [^99m^Tc]Tc-PSMA-I&S at 6 h p.i. in male LNCaP tumor-bearing CB17-SCID mice. Data are expressed as a percentage of the injected dose per gram (% ID/g), mean ± standard deviation (*n* = 4–5). gl.: gland; submand.: submandibular

Comparative biodistribution studies in LNCaP tumor-bearing mice at 6 h p.i. were performed for all four radiotracers (Fig. 3, Additional file 1: Table S3).

**Fig. 4:**
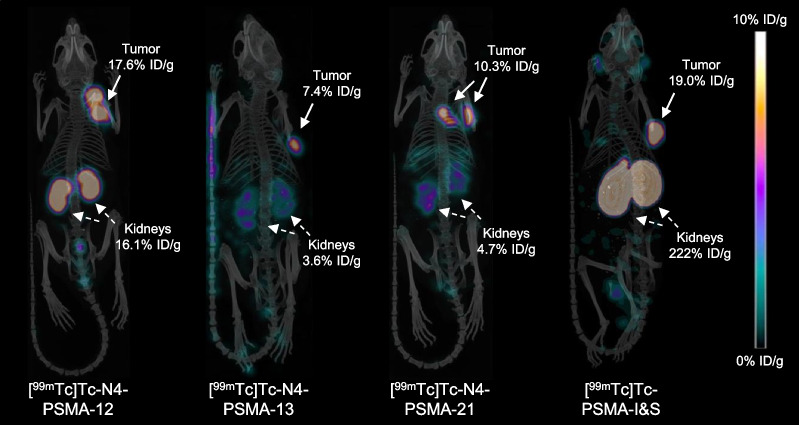
Static µSPECT/CT images (maximum intensity projections) of ^99m^Tc-labeled N4-PSMA-derivatives and [^99m^Tc]Tc-PSMA-I&S in LNCaP tumor-bearing mice. Animals were sacrificed at 6 h p.i. and imaged directly after blood collection for 45 min on a VECTor4 small-animal SPECT/PET/OI/CT. Tracer uptake in tumor and kidneys (in percent of the injected dose/gram, (% ID/g)) was determined from subsequent biodistribution studies

Among the three N4-bearing radioligands, a similar distribution profile with high uptake in the tumor (11.0–13.0% ID/g), varying but moderate activity levels in the kidneys and efficient clearance from blood and background tissue was observed (see also µSPECT/CT-scans in Fig. 4). [^99m^Tc]Tc-N4-PSMA-12 showed slightly higher kidney retention (12.3 ± 8.0% ID/g) than [^99m^Tc]Tc-N4-PSMA-13 and [^99m^Tc]Tc-N4-PSMA-21 (6.6 ± 4.8% ID/g and 4.6 ± 1.4% ID/g, respectively) which was, however, not statistically significant (*P* > 0.81). In clear contrast to the N4-PSMA radioligands, very high activity retention in the kidney was found for [^99m^Tc]Tc-PSMA-I&S (191 ± 26% ID/g, > 15-fold higher than that of [^99m^Tc]Tc-N4-PSMA-12). Significantly higher activity retention after injection of [^99m^Tc]Tc-PSMA-I&S at 6 h p.i. was also present in several other organs such as lungs, spleen, adrenals and parotid gland (*P* < 0.001 each). The higher activity uptake of [^99m^Tc]Tc-PSMA-I&S in tumors, however, was less pronounced (15.6 ± 2.8% ID/g). The corresponding differences to the novel N4-PSMA radioligands were not statistically significant (*P* > 0.27). Efficient activity clearance from the blood pool was observed for all radioligands. The lowest blood activity level at 6 h p.i. was found for [^99m^Tc]Tc-N4-PSMA-12 (0.0200 ± 0.0044% ID/g), which correlates with its low log*D*_7.4_ and low PPB. Blood activity levels of [^99m^Tc]Tc-N4-PSMA-13, [^99m^Tc]Tc-N4-PSMA-21 and [^99m^Tc]Tc-PSMA-I&S were increased by 1.6-, 5.4- and 3.6-fold, respectively, as compared to [^99m^Tc]Tc-N4-PSMA-12.

#### Tumor-to-background ratios (TBR)

**Fig. 5:**
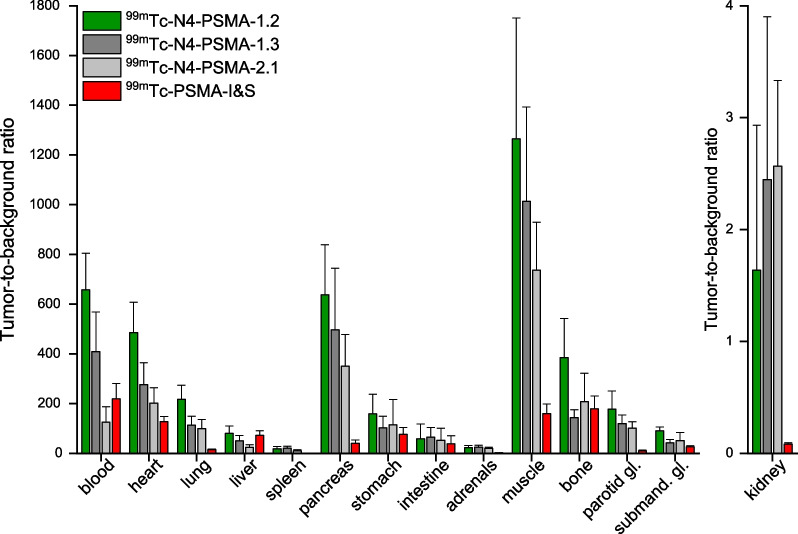
Tumor-to-background ratios (TBR) of ^99m^Tc-labeled N4-PSMA compounds and [^99m^Tc]Tc-PSMA-I&S at 6 h p.i. in male LNCaP tumor-bearing CB17-SCID mice. Data are given as mean ± standard deviation (*n* = 4–5). Mean values were determined from TBR calculated for individual animals. gl.: gland; submand.: submandibular

As depicted in Fig. 5, [^99m^Tc]Tc-N4-PSMA-12 showed the highest TBR among all radioligands throughout the majority of organs. It is noteworthy, that in direct comparison with [^99m^Tc]Tc-PSMA-I&S, [^99m^Tc]Tc-N4-PSMA-12 showed increased TBR for every analyzed organ and blood, thus demonstrating its notably superior clearance properties. In particular, a threefold T/blood-ratio (658 ± 147 vs 219 ± 62) and an even 20-fold T/kidney-ratio (1.64 ± 1.29 vs 0.08 ± 0.01) represent important advances towards optimized pharmacokinetics. Although less pronounced, improvements were also found for the TBR of [^99m^Tc]Tc-N4-PSMA-13 and [^99m^Tc]Tc-N4-PSMA-21. A tabular overview of TBR is provided in Additional file 1: Table S4.

## Discussion

With the N4-PSMA ligand design we aimed to create a versatile structural platform that ensures high PSMA affinity and reliable complexation of technetium-99m while also allowing for flexible modifications to adjust the pharmacokinetic profile of the entire ligand. To reach this goal we followed our expertise acquired in the development of theranostic rhPSMA ligands, where the combination of an EuE binding motif and a SiFA-moiety addressing the remote arene binding site has led to a series of highly potent PSMA tracers for imaging and therapy [23, 24]. To retain high affinity, while ensuring low lipophilicity of the new tracers, a less lipophilic SiOH-moiety (formally a hydrolyzed SiFA) was preferred. For complexation of technetium-99m a tetraamine (N4) chelator was chosen, as this chelating system exhibits excellent in vivo stability and confers high hydrophilicity to peptidic radioligands [29, 30]. A further advantage over N_3_S-based chelating systems such as mercaptoacetyltriserine is the absence of chemically reactive thiol-groups which might limit the shelf-live of radioligand precursors, an oftentimes neglected aspect in early radioligand development. Finally, the incorporation of a variable amino acid afforded three novel ligands with distinct properties in vitro and in vivo.

Slow whole-body clearance and in part hepatobiliary excretion of [^99m^Tc]Tc-PSMA-I&S is assumed to be caused by high PPB and increased lipophilicity compared to other PSMA-addressing radiometal chelates [15]. Therefore, a main focus in the design of the novel ligands was set on reduced lipophilicity and PPB. Fortunately, these objectives were met by all three N4-PSMA compounds. As expected, the intrinsic lipophilicity of the variable amino acid (log*P*: Phe > (4-NH_2_)-Phe > Glu) [31] translated into similar trends for lipophilicity and PPB of the corresponding radioligands (log*D*_*7.4*_ and PPB: [^99m^Tc]Tc-PSMA-I&S > [^99m^Tc]Tc-N4-PSMA-13 > [^99m^Tc]Tc-N4-PSMA-21 > [^99m^Tc]Tc-N4-PSMA-12). Especially [^99m^Tc]Tc-N4-PSMA-12 combines a favorable lipophilicity, comparable to diagnostic rhPSMA compounds [23], and a PPB that is significantly lower than for [^99m^Tc]Tc-PSMA-I&S, [^68^Ga]Ga/[^177^Lu]Lu-PSMA-I&F [32] or [^99m^Tc]Tc-EuK-(SO_3_)Cy5-mas_3_ [25] (all ligands developed for PSMA-guided surgery) and comparable to fast-clearing [^177^Lu]Lu-PSMA-617 [33]. In agreement with many prior studies [25–27], these findings underline how the pharmacokinetically relevant properties of a radioligand can be shaped in a rational fashion by thoughtful structural modifications.

Even though [^99m^Tc]Tc-PSMA-I&S showed favorable dosimetry in patients [21] and application in RGS suggests superiority over conventional salvage surgery [17], we have to admit that [^99m^Tc]Tc-PSMA-I&S is not yet the optimal radioligand for RGS. This is exemplified by a study among 31 patients by Maurer et al. who reported successful resection of all lesions detected on prior PSMA-PET and even additional lesions as small as 3 mm [13]. However, the same study revealed that in 12 of 86 resected tissue specimens that were classified PSMA-negative according to γ-probe measurements, histochemical analysis discovered previously unidentified metastatic lesions.

The obvious need for higher sensitivity in RGS is, from a radiopharmaceutical perspective, a need for an optimized radioactive probe providing higher TBR during surgery. Thus, to assess TBR of our novel ^99m^Tc-labeled N4-PSMA ligands, biodistribution studies at 6 h p.i. were performed. This rather long distribution time comes with the limitation of scarce comparability with literature, where mostly time-points of 1 h or 4 h were investigated. However, RGS is performed around 20–24 h p.i. in patients [13, 19, 36] and, based on the rule of thumb of approximately fourfold faster metabolism in mice compared to men, we chose a distribution time of 6 h to simulate TBR at the time of surgery as accurately as possible. In that context, our preclinical findings on the in vivo performance of [^99m^Tc]Tc-N4-PSMA ligands represent a remarkable development. While similar high uptake in LNCaP-xenografts was observed at 6 h p.i., the clearance of the novel ligands from most background tissue was significantly improved compared to [^99m^Tc]Tc-PSMA-I&S. In addition, drastically and favorably reduced kidney retention compared to [^99m^Tc]Tc-PSMA-I&S was found to be a common feature of the new ligands, which can probably be attributed to their shared molecular scaffold derived from rhPSMA-compounds [23, 24], while being pronounced by the use of the hydrolyzed SiFA moiety. This finding is of particular interest, as high renal activity accumulation may interfere with accurate lesion detection during RGS [36]. Nevertheless, clinical studies are necessary to validate whether our preclinical findings also apply to the human situation. To an even greater extend, incomplete clearance of [^99m^Tc]Tc-PSMA-I&S from the blood pool and thus a suboptimal T/blood-ratio can affect the accuracy of RGS [15]. In this regard, the 3.6-fold decreased blood activity at 6 h p.i. represents another distinct advantage of [^99m^Tc]Tc-N4-PSMA-12 and emphasizes the positive influence of the negatively charged amino acid d-Glu towards accelerated clearance kinetics. Compared to [^99m^Tc]Tc-N4-PSMA-12, slightly higher uptake in blood and several background organs such as heart, lung, liver, parotid gland and submandibular gland were observed for [^99m^Tc]Tc-N4-PSMA-13 and [^99m^Tc]Tc-N4-PSMA-21, which can be attributed to the incorporation of the aromatic amino acids d-Phe and d-(4-NH_2_)Phe conferring higher lipophilicity and plasma protein binding to these compounds. A further possible limitation of [^99m^Tc]Tc-PSMA-I&S is background activity in the bowel hampering the detection of lesions with low signal strength during surgery. In this regard, when completely ignoring the significantly improved T/kidney ratio of [^99m^Tc]Tc-N4-PSMA-12 and when taking into account only the hepatic and intestinal uptake of [^99m^Tc]Tc-PSMA-I&S and [^99m^Tc]Tc-N4-PSMA-12 (no statistically significant difference observed, *P* > 0.19 and *P* > 0.36, respectively), we would expect at least similar performance of [^99m^Tc]Tc-PSMA-I&S and [^99m^Tc]Tc-N4-PSMA-12 in men. Beyond that, the finding that TBR of blood and all analyzed organs (see Fig. 5) obtained with [^99m^Tc]Tc-N4-PSMA-12 are higher than that of [^99m^Tc]Tc-PSMA-I&S clearly demonstrates the superior pharmacokinetic profile of that radioligand. Finally, as the only apparent advantage of [^99m^Tc]Tc-N4-PSMA-13 and [^99m^Tc]Tc-N4-PSMA-21 over [^99m^Tc]Tc-N4-PSMA-12, namely a slightly decreased kidney retention, was not statistically significant (*P* > 0.81), and based on the aforementioned superior overall pharmacokinetic profile of [^99m^Tc]Tc-N4-PSMA-12, the latter was identified as most promising compound.

Besides [^99m^Tc]Tc-PSMA-I&S, the feasibility of PSMA-targeted RGS using [^99m^Tc]Tc-MIP-1404, a tracer originally developed for SPECT-imaging, was recently described in a small cohort of nine patients [34]. In a preclinical evaluation by Hillier et al. this tracer had shown a tissue distribution profile at 4 h p.i. which is highly similar to our data for [^99m^Tc]Tc-N4-PSMA-12 (see Additional file 1: Fig. S2) [37]. Surprisingly and in contrast to the pharmacokinetics observed in mice, biodistribution studies in patients revealed high hepatic uptake and delayed clearance for [^99m^Tc]Tc-MIP-1404 [35] indicating limited transferability of preclinical data to the human situation for this radioligand. Consistent with the similar patient dosimetry reported for [^99m^Tc]Tc-PSMA-I&S and [^99m^Tc]Tc-MIP-1404 [21, 35], the latter yielded comparable results in the recent RGS study [34]. This example underlines not only the need for an optimized radioactive probe in PSMA-targeted RGS but also that, ultimately, clinical studies will be needed to assess whether the promising performance of [^99m^Tc]Tc-N4-PSMA-12 in mice will also translate into improved outcomes of RGS in patients.

**Fig. 6:**
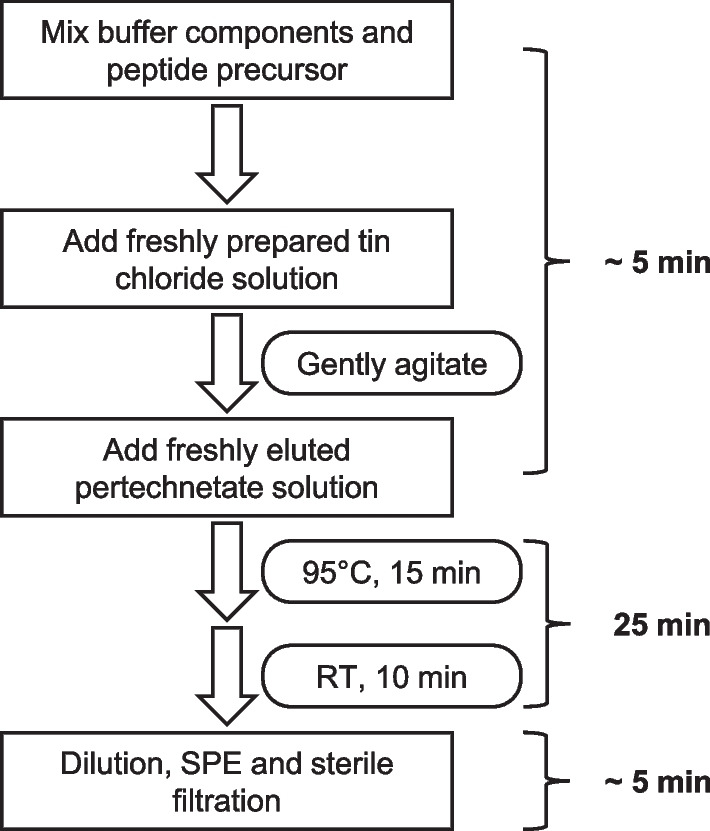
Suggested work-flow for clinical production of [^99m^Tc]Tc-N4-PSMA-12. Patient scale production is completed within 35 min post generator elution under non-optimized laboratory conditions. Optimization for clinical routine synthesis will further reduce the time of production. RT: room temperature; SPE: solid phase extraction

To facilitate future clinical translation, we developed a protocol for GMP-production of [^99m^Tc]Tc-N4-PSMA-12 (see Fig. 6). As the simple, reliable, one-step process complies with basic nuclear medicine infrastructure and established clinical workflows and procedures, it provides an important basis for widespread availability of [^99m^Tc]Tc-N4-PSMA-12 and may contribute to minimize the logistic hurdle for a first clinical application.

In summary, the rational design, development and comparative preclinical evaluation of three novel ^99m^Tc-labeled PSMA inhibitors resulted in the identification of [^99m^Tc]Tc-N4-PSMA-12 as lead candidate. Compared to [^99m^Tc]Tc-PSMA-I&S, this novel radioligand showed a significantly improved pharmacokinetic profile, 3.6-fold reduced blood activity, 15-fold decreased kidney activity and improved TBR for blood and all organs. Furthermore, a clinically suited radiolabeling process is suggested rendering [^99m^Tc]Tc-N4-PSMA-12 a highly attractive candidate for clinical application in RGS.

## Conclusion

Based on the promising data presented in this preclinical work, a first clinical study with [^99m^Tc]Tc-N4-PSMA-12 is highly warranted. It can be expected that this tracer has the potential to overcome current limitations obtained with [^99m^Tc]Tc-PSMA-I&S and thus might pave the way towards a broader future use of PSMA-targeted RGS in the therapy of patients with biochemical recurrent PCa.

## Supplementary Information

**Additional file 1.** Supplementary information.

## Data Availability

The datasets used and analyzed during the current study are available from the corresponding author on reasonable request.

## Abbreviations

HPLC: High performance liquid chromatography
IBA-KuE: (((*S*)-1-Carboxy-5-(4-(iodo)benzamido)pentyl)carbamoyl)-l-glutamic acid
LNM: Lymph node metastases
mCRPC: Metastatic castration resistant prostate cancer
N4: Tetraamine/6-carboxy-1,4,8,11-tetraazaundecane
PBS: Phosphate buffered saline
PCa: Prostate cancer
PET: Positron emission tomography
PPB: Plasma protein binding
PSMA: Prostate-specific membrane antigen
RCP: Radiochemical purity
RGS: Radioguided surgery
RP: Reversed phase
SD: Standard deviation
SiFA: Silicon fluoride acceptor
sLND: Sentinel lymph node dissection
SPECT: Single photon emission computed tomography
TBR: Tumor-to-background ratio
TLC: Thin layer chromatography
UV: Ultra-violet
