# Supplementary material for: Synthesis and preclinical evaluation of novel 99mTc-labeled PSMA ligands for radioguided surgery of prostate cancer
Source: EJNMMI Res. 2023 Jan 16;13:2. doi: 10.1186/s13550-022-00942-7 (PMC9842843; doi:10.1186/s13550-022-00942-7)
Supplement: Supplementary file 1 — Additional file 1. Supplementary information. [file 13550_2022_942_MOESM1_ESM.docx]

**Synthesis and Preclinical Evaluation of Novel ^99m^Tc-labeled PSMA Ligands for Radioguided Surgery of Prostate Cancer**

**----- Supplementary Information -----**

Jan-Philip Kunert, Max Müller, Thomas Günther, León Stopper, Nicole Urtz-Urban, Roswitha Beck and Hans-Jürgen Wester

Technical University of Munich (TUM), Germany; TUM Department of Chemistry, Chair of Pharmaceutical Radiochemistry, Garching, Germany.

**Corresponding author:**

Jan-Philip Kunert, M.Sc.

Phone: +49.89.289.12203

Fax: +49.89.289.12204

Email: jan-philip.kunert@tum.de

Technical University of Munich,

Chair of Pharmaceutical Radiochemistry,

Walther-Meißner-Str. 3

85748 Garching

GERMANY

**General Information**

Protected amino acids for peptide synthesis were purchased from Carbolution (St. Ingbert, Germany) and Iris Biotech (Marktredwitz, Germany). The 2-Chlorotrityl chloride polystyrene (TCP) resin was obtained from Sigma-Aldrich (Steinheim, Germany). Solvents and all other organic and inorganic reagents were purchased from Alfa Aesar (Karlsruhe, Germany), Fluorochem (Hadfield, United Kingdom), Sigma-Aldrich (Steinheim, Germany) or VWR (Darmstadt, Germany) and used without further purification. Radioactive [^99m^Tc]TcO_4_^-^ was obtained from a Ultra-Technekow FM 2 (15 - 43.00 GBq) generator (Curium, Petten, Netherlands).

Solid phase peptide synthesis (SPPS) was carried out manually in syringe reactors for peptide synthesis (Carl Roth, Karlsruhe, Germany) using a MX-RD-Pro syringe shaker from SCILOGEX (Rocky Hill, United States). Analytical and preparative reversed-phase high-performance liquid chromatography (*RP*-HPLC) was performed using Shimadzu gradient systems (Shimadzu, Neufahrn, Germany) each equipped with a SPD-20A UV/Vis-detector (detection at λ = 220 nm) and LC-20AD solvent pumps. Eluents for all chromatographic procedures were water (solvent A, 0.1% TFA (*v*/*v*)) and acetonitrile (solvent B, 0.1% TFA (*v*/*v*), 2% or 5% water (*v*/*v*) in analytical or preparative procedures, respectively). For analytical measurements a MultoKrom 100-5 C18 column (150 mm x 4.6 mm, CS Chromatographie-Service, Langerwehe, Germany) was used at a constant flow rate of 1 mL/min. Preparative *RP*‑HPLC was performed on a MultoKrom 100-5 C18 column (250 mm x 20 mm, CS Chromatographie-Service) applying a constant flow rate of 10 mL/min. Reversed‑phase high performance flash chromatography (*RP*‑HPFC) was performed on an SP HPFC system with SNAP cartridges (KP‑C18‑HS, 12 g) from Biotage (Charlottesville, United States) applying water (solvent A, 0.1% TFA (*v*/*v*)) and acetonitrile (solvent B, 0.1% TFA (*v*/*v*)) as eluents. Electrospray ionization (ESI) mass spectra and atmospheric pressure chemical ionization (APCI) mass spectra for compound characterization were acquired on an expression^L^ CMS mass spectrometer from Advion (Harlow, United Kingdom). ^1^H‑NMR-spectra were acquired on an AVHD 400 from Bruker (Billerica, United States) at 300 K. Chemical shifts (*δ*) are given in parts per million (ppm), spectra are calibrated to the residual ^1^H solvent signal of DMSO‑*d*_6_ at 2.50 ppm and signal multiplicities are described as: s = singlet, m = multiplet.

Analytical and preparative radio *RP*‑HPLC was performed on a Shimadzu system equivalent as stated above and additionally equipped with a SIL‑20A HAT autosampler using a MultoKrom 100-5 C18 column (125 mm x 4.6 mm) from CS Chromatographie-Service at a constant flow rate of 1 mL/min. A HERM LB 500 NaI scintillation detector (Berthold Technologies, Bad Wildbad, Germany) was connected to the outlet of the UV-photometer for the detection of radioactivity. Radio thin layer chromatography (TLC) was performed on iTLC-SG stripes (Agilent Technologies, Waldbronn, Germany) using butanone or NH_4_OAc (1 m in water) with DMF (1/1 (*v*/*v*)) as mobile phase for quantification of free [^99m^Tc]TcO_4_^-^ or colloidal technetium-99m, respectively. Radio-TLC stripes were analyzed using a Scan-RAM Radio-TLC detector from LabLogic Systems (Sheffield, United Kingdom). Activity quantification of radioactive probes was carried out using a 2480 WIZARD^2^ automatic gamma counter (PerkinElmer, Waltham, United States).

Centrifuges used for the determination of lipophilicity and binding to human plasma were a HERAEUS Pico 17 and a HERAEUS Megafuge 16R, respectively (Thermo Scientific, Osterode, Germany).

**Solution state synthesis of building blocks for SPPS**

*(tBu)_2_EuE(tBu)*:

The *tert*‑butyl protected Glu‑urea‑Glu binding motive was synthesized in analogy to the synthesis of *tert*‑butyl protected Lys‑urea‑Glu reported in literature [1, 2].

*4-(Di-tert-butylhydroxysilyl)benzoic acid (SiOH-BA):*

4-(Di-tert-butylhydroxysilyl)benzoic acid (SiOH‑BA) was obtained by hydrolysis of 4-(Di-tert-butylfluorosilyl)benzoic acid (SiFA-BA). The latter was synthesized according to a published procedure [3]. To a stirring solution of SiFA-BA (114 mg, 403 µmol, 1.0 eq.) in DMF (4 mL) a solution of KOH (113 mg, 2013 µmol, 5.0 eq.) in TP-water (1 mL) was added at room temperature and stirred for 1 h. The solution was acidified (pH 4‑5) by addition of 2.013 mL 1 m HCl_(aq)_ and extracted with diethyl ether (5x 5 mL). The combined organic phases were dried over MgSO_4_ and solvents were evaporated *in vacuo*. Residual DMF was removed via lyophilization to obtain the product as colorless, amorphous solid (96%).

RP-HPLC (50‑100% B in 15 min): t_R_ = 5.8 min, K’ = 2.90. Calculated monoisotopic mass (C_15_H_24_O_3_Si): 280.2; found: m/z (APCI) = 279.0 [M-H]^-^.

*N,N’,N’’,N’’’‑Tetrakis(tert‑butyloxycarbonyl)‑6‑carboxy-1,4,8,11-tetraazaundecane ((tBu)_4_N_4_):*

*N*‑Boc‑ethylenediamine (4.0 eq.) was slowly added to a solution of 3‑bromo‑2‑(bromomethyl)-propionic acid (1.0 eq.) in THF (25 mL/mmol) and stirred for 24 h at room temperature. The solvent was removed *in vacuo* and the crude residue dissolved in acetone/H_2_O (1/1 (*v*/*v*), 25 mL/mmol). The solution was cooled to 0°C and triethylamine (3.0 eq.) was added. After 5 min di‑*tert*‑butyl dicarbonate (4.0 eq.) was added and the stirring mixture was left to warm up to room temperature within 15 h. Solvents were removed *in vacuo*, the raw product was purified via *RP*‑HPFC (35-71% B in 15min) and the desired product was obtained as colorless, amorphous solid.

ESI‑MS: calculated monoisotopic mass (C_28_H_52_N_4_O_10_): 604.4; found: m/z (ESI) = 605.5 [M+H]^+^.

^1^H‑NMR (400 MHz, DMSO‑*d*_6_) *δ* = 7.17‑6.19 (m, 2H, NH), 3.28‑3.17 (m, 6H, CH_2_), 3.10‑2.95 (m, 6H, CH_2_), 2.94‑2.90 (m, 1H, CH), 1.38 (s, 18H, CH_3_), 1.36 (s, 18H, CH_3_).

**Synthesis of PSMA ligands**

Chemical synthesis of novel N4-bearing PSMA-ligands was carried out via Fmoc-based standard solid phase peptide synthesis (SPPS). Reference compound PSMA-I&S was synthesized as described earlier [4]. Purity of all labeling precursors was determined via *RP*-HPLC (UV-detection at 220 nm) and was >98% in all cases.

*General procedures (GP) for solid phase peptide synthesis*:

All synthetic steps were carried out in a syringe reactor for peptide synthesis at room temperature. After each coupling or deprotection step, the resin was thoroughly washed with DMF (5 mL/g resin) six or eight times, respectively.

*TCP-resin loading (GP1)*: The amino acid (2.0 eq.) and DIPEA (3.75 eq.) are dissolved in DMF (5 mL/g resin) and added to the TCP resin. After 2.5 h, methanol (2 mL/g resin) is added for capping of remaining trityl chloride groups. Subsequently, the resin is washed thoroughly with DMF (6x 5 mL/g resin), DCM (3x 5 mL/g resin) and methanol (3x 5 mL/g resin) and dried *in vacuo*. The loading $l$ of the amino acid is determined by the equation

$$l\left[ \frac{mmol}{g} \right]=\frac{\left( m_{2}-m_{1} \right)\cdot1000}{\left( M_{AA}-M_{HCl} \right)\cdot m_{2}}$$

with $m_{1}$ = mass of unloaded resin [g], $m_{2}$ = mass of loaded resin [g], $M_{AA}$ = molecular weight of amino acid [g/mol] and $M_{HCl}$ = molecular weight of HCl [g/mol].

*On-resin amide bond formation (GP2)*: For the conjugation of Fmoc-protected amino acids and other building blocks, their carboxylic acid functionality is preactivated by addition of TBTU (2.0 eq.), HOAt (2.0 eq.) and DIPEA (6.0 eq.) in DMF. After 5 min the solution is added to the resin and left to react for 2.5 h (differing coupling times are mentioned in the synthesis protocol). Coupling of Fmoc-*D*-Dap(Dde)-OH is performed with 2,4,6‑trimethylpyridine (6.7 eq.) as base instead of DIPEA to prevent racemization.

*On-resin Fmoc-deprotection (GP3)*: Deprotection of Fmoc-protecting groups is achieved by addition of 20% piperidine in DMF (8 mL/g resin) for 5 min and subsequently for 15 min.

*N4-PSMA ligands*:

Fmoc-*D*-Orn(Dde)-OH was loaded to the TCP resin as first building block according to GP1, and after subsequent Fmoc cleavage (GP3), (*t*BuO)EuE(O*t*Bu)_2_ was conjugated for 4.5 h (GP2). Deprotection of Dde was carried out using a solution of 2% hydrazine monohydrate in DMF (5 mL/g resin) for 20 min. Subsequently, a solution of succinic anhydride (7 eq.) and DIPEA (7 eq.) in DMF was added and left to react for 2.5 h. The resin bound carboxylate was then preactivated by addition of TBTU (2.0 eq.), HOAt (2.0 eq.) and DIPEA (6.0 eq.) in DMF for 30 min and Fmoc-*D*-Lys-OtBu (2.0 eq.) in DMF was added for conjugation for 2.5 h. Subsequent Fmoc deprotection (GP3) was followed by conjugation of Fmoc-*D*-Dap(Dde)-OH (GP2). Orthogonal deprotection of Dde was carried out using hydroxylamine hydrochloride (1.26 g/g resin) and imidazole (0.92 g/g resin) in a mixture of DMF (1 mL/g resin) and NMP (5 mL/g resin) for 3.5 h. Subsequently, SiOH-BA was conjugated (GP2) and the remaining Fmoc protecting group was cleaved according to GP3. In the following step, either Fmoc‑*D*‑Glu(O*t*Bu)‑OH (in N4-PSMA-12), Fmoc‑*D*‑Phe‑OH (in N4-PSMA-13) or Fmoc‑*D*‑Phe(4-NHBoc)‑OH (in N4-PSMA-21) was coupled according to GP2. After subsequent Fmoc-deprotection (GP3), the *tert*-butyl protected N4‑chelator was conjugated according to GP2. Cleavage from the resin and simultaneous deprotection of the ligand was performed in TFA (+2.5% TIPS, +2.5% H_2_O) for 1 h. After purification by semipreparative *RP*-HPLC, N4-PSMA-12, N4-PSMA-13 and N4-PSMA-21 were obtained as colorless, amorphous solids in yields of 29%, 25% and 21%, respectively (yields refer to the amount of substance of resin‑bound Fmoc‑*D*‑Orn(Dde) at the start of solid phase synthesis).

N4-PSMA-12: RP-HPLC (10‑60% B in 15 min): t_R_ = 9.3 min, K’ = 3.72. Calculated monoisotopic mass (C_57_H_95_N_13_O_21_Si): 1325.7; found: m/z (ESI) = 1326.5 [M+H]^+^, 664.0 [M+2H]^2+^.

N4-PSMA-13: RP-HPLC (10‑60% B in 15 min): t_R_ = 10.2 min, K’ = 4.08. Calculated monoisotopic mass (C_61_H_97_N_13_O_19_Si): 1343.7; found: m/z (ESI) = 1344.4 [M+H]^+^, 673.0 [M+2H]^2+^.

N4-PSMA-21: RP-HPLC (10‑60% B in 15 min): t_R_ = 9.0 min, K’ = 3.60. Calculated monoisotopic mass (C_61_H_98_N_14_O_19_Si): 1358.7; found: m/z (ESI) = 1359.7 [M+H]^+^, 680.5 [M+2H]^2+^.

*IBA-KuE*:

For the synthesis of IBA-KuE, the protected binding motive (O*t*Bu)KuE(O*t*Bu)_2_ was synthesized as previously described [1]. 4-iodo-benzoic acid (6.1 mg, 24.6 µmol, 1.2 eq.) was preactivated by addition of TBTU (7.9 mg, 24.6 µmol, 1.2 eq.), HOAt (3.3 mg, 24.6 µmol, 1.2 eq.) and DIPEA (12.9 µL, 73.8 µmol, 3.6 eq.) in DMF (1 mL). After 5 min of pre-activation at room temperature, (O*t*Bu)KuE(O*t*Bu)_2_ (10.0 mg, 20.5 µmol, 1.0 eq.) in DMF (2 mL) was added and the solution was stirred overnight (21 h) at room temperature. The solvent was evaporated and upon addition of TFA (+2.5% TIPS, +2.5% H_2_O) the solution was stirred for 1 h. TFA was evaporated and the crude product dissolved in DMF. After purification by semi-preparative *RP*-HPLC (30-45% B in 20 min) the product was obtained as colorless, amorphous solid (48%).

RP-HPLC (20‑40% B in 20 min): t_R_ = 11.0 min, K’ = 7.68. Calculated monoisotopic mass (C_19_H_24_IN_3_O_8_): 549.1; found: m/z (ESI) = 550.2 [M+H]^+^.

**Radiosynthesis of [^99m^Tc]Tc‑N4‑PSMA‑12 at patient scale**

In a 10 mL glass vial, 15 nmol N4-PSMA-12 (20 µg, 0.5 mm in DMSO) were added to a mixture of 0.05 m Na_2_HPO_4_ (250 µL, in TP-water, pH 9.25) and 0.1 m disodium citrate sesquihydrate (30 µL, in TP-water) in saline. After addition of a freshly prepared solution of SnCl_2_ (10 µL, 1 mg/mL in ethanol), [^99m^Tc]TcO_4_^-^ in saline was added and the labeling solution (final volume 2‑5 mL) was heated to 95°C for 15 min. The labeling solution was left to cool for 10 min and, subsequently, quality control was performed using radio-TLC and radio-*RP*-HPLC.

**Radiosynthesis of [^99m^Tc]Tc‑PSMA‑I&S**

Labeling of PSMA-I&S was carried out using 2 nmol of peptide precursor in a kit formulation as described by Robu et al [4]. After addition of [^99m^Tc]TcO_4_^-^ (40 MBq/nmol) in 500 µL saline the solution was heated to 95°C for 20 min. Subsequently, 10 µL of 1 m sodium ascorbate (PBS) was added and quality control was performed using radio-TLC and radio-*RP*-HPLC.

**Radioiodination of [^125^I]IBA‑KuE**

The synthesis of the protected stannyl-precursor, radioiodination and deprotection yielding [^125^I]IBA-KuE was carried out as previously described [1]. Purification of the crude labelling product was performed using preparative radio-*RP*-HPLC with a gradient of 20‑40% B in 20 min.

**Analytical data of PSMA inhibitors labeled with technetium‑99m or iodine‑125**

[^99m^Tc]Tc-N4-PSMA-12: radio-RP-HPLC (10‑70% B in 15 min): t_R_ = 9.1 min, K’ = 6.36.

[^99m^Tc]Tc-N4-PSMA-13: radio-RP-HPLC (10‑70% B in 15 min): t_R_ = 10.0 min, K’ = 6.98.

[^99m^Tc]Tc-N4-PSMA-21: radio-RP-HPLC (10‑70% B in 15 min): t_R_ = 8.6 min, K’ = 5.99.

[^99m^Tc]Tc-PSMA-I&S: radio-RP-HPLC (10‑70% B in 15 min): t_R_ = 8.2 min, K’ = 5.76.

[^125^I]IBA-KuE: radio-RP-HPLC (20‑40% B in 20 min): t_R_ = 11.0 min, K’ = 7.68.

**Supplementary Table 1:** Detailed data on ^99m^Tc-labeling of N4-PSMA-12 at patient scale. The volume of the labeling solution (V), the applied activity (A) as well as the amount of free [^99m^Tc]TcO_4_^-^ and colloidal technetium-99m, as determined via radio‑TLC, and the resulting radio chemical purity (RCP) are given for single labeling experiments. Mean values and standard deviation (SD, n = 10) are given for the whole data set.

| **entry** | **V**  **[mL]** | **A**  **[MBq]** | **Free [^99m^Tc]TcO_4_^−^ [%]** | **Colloidal Tc‑99m [%]** | **RCP**  **[%]** |
| --- | --- | --- | --- | --- | --- |
| 1 | 5.00 | 194 | 0.21 | 0.85 | 98.94 |
| 2 | 5.00 | 810 | 0.40 | 0.44 | 99.16 |
| 3 | 5.00 | 542 | 0.24 | 2.21 | 97.55 |
| 4 | 5.00 | 502 | 0.17 | 1.98 | 97.85 |
| 5 | 5.00 | 760 | 0.30 | 1.78 | 97.92 |
| 6 | 3.18 | 634 | 0.23 | 1.12 | 98.65 |
| 7 | 2.66 | 470 | 0.23 | 1.02 | 98.75 |
| 8 | 3.02 | 388 | 0.31 | 0.64 | 99.05 |
| 9 | 2.00 | 727 | 0.97 | 0.73 | 98.30 |
| 10 | 4.00 | 501 | 0.28 | 1.31 | 98.41 |
| **mean** | **3.99** | **553** | **0.33** | **1.21** | **98.46** |
| **SD** | **1.18** | **187** | **0.23** | **0.60** | **0.55** |

**Supplementary Table 2:** Lipophilicity of the three novel ^99m^Tc-labeled N4-PSMA compounds and ^99m^Tc-labeled PSMA-I&S expressed as partition coefficient (log*D_7.4_*) using the *n*‑octanol/PBS (pH 7.4) distribution system (n = 8), binding affinity towards PSMA (inverse IC_50_ (nM), 1 h, 4°C, n = 3), PSMA-mediated internalization by LNCaP cells (1 h, 37°C, n = 3) as a percentage of the radiolabeled reference ([^125^I]IBA)KuE) and plasma protein binding (PPB, determined by a ultrafiltration method, n = 6) of ^99m^Tc-labeled N4-PSMA compounds and [^99m^Tc]Tc‑PSMA-I&S.

| **compound** | **[^99m^Tc]Tc-N4‑PSMA-12** | **[^99m^Tc]Tc-N4‑PSMA-13** | **[^99m^Tc]Tc-N4‑PSMA-21** | **[^99m^Tc]Tc-PSMA‑I&S** |
| --- | --- | --- | --- | --- |
| log*D*_7.4_ | −3.35 ± 0.05 | −2.78 ± 0.05 | −3.13 ± 0.05 | −2.61 ± 0.08 |
| IC_50, inv._ (nM) | 11.02 ± 2.80 | 9.98 ± 0.99 | 11.07 ± 1.42 | 11.78 ± 1.89 |
| internalization  (% IBA-KuE) | 311 ± 16 | 164 ± 15 | 180 ± 4 | 240 ± 13 |
| PPB (%) | 55.1 ± 2.5 | 88.5 ± 1.5 | 66.0 ± 2.3 | 94.4 ± 0.7 |

**Supplementary Table 3:** Numeric data of the biodistribution of ^99m^Tc-labeled N4-PSMA compounds and ^99m^Tc-labeled PSMA-I&S at 6 h p.i. in male LNCaP tumor-bearing CB17‑SCID mice. Data are expressed as percentage of the injected dose per gram (% ID/g), mean ± standard deviation (SD, n = 4‑5).

|  | **[^99m^Tc]Tc-N4‑PSMA-12**  **(n = 5)** | | **[^99m^Tc]Tc-N4‑PSMA-13**  **(n = 5)** | | **[^99m^Tc]Tc-N4‑PSMA-21**  **(n = 4)** | | **[^99m^Tc]Tc-PSMA‑I&S**  **(n = 4)** | |
| --- | --- | --- | --- | --- | --- | --- | --- | --- |
| **uptake in % iD/g** | **mean** | **SD** | **mean** | **SD** | **mean** | **SD** | **mean** | **SD** |
| blood | 0.0200 | 0.0044 | 0.0320 | 0.0100 | 0.1074 | 0.0623 | 0.0728 | 0.0072 |
| heart | 0.0274 | 0.0083 | 0.0456 | 0.0090 | 0.0568 | 0.0128 | 0.1246 | 0.0252 |
| lung | 0.0613 | 0.0177 | 0.1108 | 0.0186 | 0.1250 | 0.0578 | 1.0378 | 0.0612 |
| liver | 0.1796 | 0.0890 | 0.2618 | 0.0556 | 0.5752 | 0.3652 | 0.2215 | 0.0234 |
| spleen | 0.8928 | 0.5700 | 0.6314 | 0.1819 | 1.3064 | 0.7969 | 31.8636 | 6.0507 |
| pancreas | 0.0206 | 0.0031 | 0.0270 | 0.0053 | 0.0335 | 0.0099 | 0.4040 | 0.0639 |
| stomach | 0.0988 | 0.0506 | 0.1348 | 0.0441 | 0.1458 | 0.0956 | 0.2306 | 0.1018 |
| intestine | 0.5391 | 0.4453 | 0.2661 | 0.1637 | 1.1034 | 1.7380 | 0.6415 | 0.3960 |
| kidney | 12.2924 | 8.0491 | 6.5974 | 4.7823 | 4.5811 | 1.4363 | 191.560 | 25.7140 |
| adrenals | 0.6570 | 0.2747 | 0.5170 | 0.0912 | 0.5759 | 0.1443 | 13.5354 | 2.9883 |
| muscle | 0.0110 | 0.0041 | 0.0127 | 0.0018 | 0.0153 | 0.0027 | 0.1020 | 0.0290 |
| bone | 0.0365 | 0.0136 | 0.0862 | 0.0139 | 0.0828 | 0.0778 | 0.0907 | 0.0157 |
| tumor | 13.0231 | 3.8549 | 12.5347 | 4.0835 | 11.0155 | 2.2227 | 15.6183 | 2.8033 |
| parotid gland | 0.0804 | 0.0339 | 0.1056 | 0.0262 | 0.1105 | 0.0199 | 1.7348 | 0.5573 |
| submandibular gland | 0.1450 | 0.0431 | 0.3069 | 0.0642 | 0.3009 | 0.2151 | 0.6196 | 0.0695 |

**Supplementary Table 4:** Tumor-to-background ratios of ^99m^Tc-labeled N4-PSMA compounds and ^99m^Tc-labeled PSMA-I&S at 6 h p.i. in male LNCaP tumor-bearing CB17‑SCID mice. Data are given as mean ± standard deviation (SD, n = 4‑5). Mean values were determined from tumor-to-organ ratios calculated for individual animals.

| **tumor-to-background** | **[^99m^Tc]Tc-N4‑PSMA-12**  **(n = 5)** | | **[^99m^Tc]Tc-N4‑PSMA-13**  **(n = 5)** | | **[^99m^Tc]Tc-N4‑PSMA-21**  **(n = 4)** | | **[^99m^Tc]Tc-PSMA‑I&S**  **(n = 4)** | |
| --- | --- | --- | --- | --- | --- | --- | --- | --- |
| **ratio** | **mean** | **SD** | **mean** | **SD** | **mean** | **SD** | **mean** | **SD** |
| blood | 657.74 | 146.75 | 409.29 | 158.60 | 125.55 | 62.05 | 218.97 | 62.09 |
| heart | 484.90 | 122.64 | 276.38 | 87.34 | 201.50 | 62.05 | 127.22 | 20.53 |
| lung | 216.68 | 56.82 | 113.30 | 35.32 | 99.35 | 36.86 | 15.01 | 2.29 |
| liver | 79.92 | 30.33 | 50.17 | 21.50 | 23.75 | 10.51 | 71.64 | 19.02 |
| spleen | 17.89 | 9.02 | 20.57 | 7.45 | 10.32 | 4.39 | 0.49 | 0.06 |
| pancreas | 637.21 | 201.56 | 496.73 | 247.35 | 350.74 | 126.56 | 40.19 | 13.36 |
| stomach | 158.87 | 78.55 | 101.82 | 47.12 | 114.49 | 101.37 | 76.24 | 27.21 |
| intestine | 57.76 | 60.23 | 64.62 | 39.59 | 52.29 | 48.36 | 38.15 | 31.76 |
| kidney | 1.64 | 1.29 | 2.45 | 1.45 | 2.57 | 0.77 | 0.08 | 0.01 |
| adrenals | 22.15 | 9.04 | 24.50 | 8.36 | 19.85 | 4.82 | 1.24 | 0.52 |
| muscle | 1265.34 | 486.05 | 1013.12 | 379.65 | 736.44 | 193.04 | 159.28 | 39.53 |
| bone | 384.40 | 158.05 | 142.94 | 32.47 | 207.38 | 115.07 | 178.36 | 51.53 |
| parotid gland | 177.38 | 73.36 | 119.35 | 34.53 | 101.59 | 24.83 | 9.87 | 3.90 |
| submandibular gland | 90.45 | 15.58 | 41.60 | 14.26 | 50.80 | 32.33 | 25.44 | 5.00 |

**Analysis of biodistribution data of [^99m^Tc]Tc-N4-PSMA-21**

We report the following data in order to comply with the ARRIVE guidelines for the reporting of research involving animals. The biodistribution study of [^99m^Tc]Tc-N4-PSMA-21 was carried out with n = 5 animals (“data set I”). All animals were healthy and did not show any signs of pain, distress or change in behavior throughout the experiment. However, as depicted in supplementary figure 1, gross deviations in the kidney uptake and elevated tumor uptake were observed for one particular animal (mouse #3) as compared to the other four animals. Kidney uptake of mouse #3 (36.17 %ID/g) exceeded the value obtained from the other four animals (4.58 ± 1.44 %ID/g, mean ± SD) 7.9-fold or by more than 20 standard deviations. In addition, tumor uptake of mouse #3 (21.92 %ID/g) exceeded the value obtained from the other four animals (11.02 ± 2.22 %ID/g, mean ± SD) 2.0-fold or by more than 4 standard deviations Due to the uncertainty, whether these huge deviations arise from an experimental error or might be related to strongly deviating metabolic processes e.g. abnormal kidney function in mouse #3, the study primarily presents and discusses the data set based on the other four animals (n = 4, “data set II”).

A thorough evaluation and comparison was, however, also carried out including mouse #3. For both, “data set I” (n = 5, including mouse #3) and “data set II” (n = 4, without mouse #3) kidney uptake of [^99m^Tc]Tc-N4-PSMA-21 was significantly reduced compared to [^99m^Tc]Tc-PSMA-I&S (both *P* < 0.001) but not statistically different from other ^99m^Tc-labeled N4-PSMA-ligands (*P* > 0.92 and *P* > 0.81 for “data set I” and “data set II”, respectively). Although mouse #3 also showed an elevated tumor uptake (see above), no statistically significant differences in tumor uptake among the ^99m^Tc-labeled PSMA-ligands evaluated herein were found, regardless whether “data set I” (*P* > 0.69) or “data set II” (*P* > 0.27) was compared to the biodistribution data of the other radioligands.

**Supplementary Figure 1:** Biodistribution of [^99m^Tc]Tc-N4-PSMA-21 in a single mouse #3 (red) showing drastically increased kidney uptake and elevated tumor uptake as compared to the other four animals depicted by “data set II” (dark green, n = 4, without mouse #3). “Data set I” (dark red, n = 5, including mouse #3) represents the biodistribution of [^99m^Tc]Tc-N4-PSMA-21 including all animals. Data are expressed as percentage of the injected dose per gram (% ID/g), mean ± standard deviation.

**Supplementary Figure 2:** Comparison of ex vivo biodistribution data of ^99m^Tc-labeled MIP-1404 at 4 h p.i. in male LNCaP tumor-bearing NCr-*nu/nu* mice with ex vivo biodistribution data of the three novel ^99m^Tc-labeled N4-PSMA compounds and ^99m^Tc-labeled PSMA-I&S at 6 h p.i. in male LNCaP tumor-bearing CB17‑SCID mice. Data are expressed as percentage of the injected dose per gram (% ID/g), mean ± standard deviation (n = 4‑5). Organs for which no data on [^99m^Tc]Tc-MIP-1404 were available in literature are indicated with an asterisk. Biodistribution data of [^99m^Tc]Tc-MIP-1404 were originally published in *JNM* (Hillier et al. ^99m^Tc-Labeled Small-Molecule Inhibitors of Prostate-Specific Membrane Antigen for Molecular Imaging of Prostate Cancer. J Nucl Med. 2013;54:1369-76. ©SNMMI)

**References**

1. Weineisen M, Simecek J, Schottelius M, Schwaiger M, Wester HJ. Synthesis and preclinical evaluation of DOTAGA-conjugated PSMA ligands for functional imaging and endoradiotherapy of prostate cancer. EJNMMI Res. 2014;4:63. doi:10.1186/s13550-014-0063-1.

2. Robu S, Schmidt A, Eiber M, Schottelius M, Gunther T, Hooshyar Yousefi B, et al. Synthesis and preclinical evaluation of novel ^18^F-labeled Glu-urea-Glu-based PSMA inhibitors for prostate cancer imaging: a comparison with ^18^F-DCFPyl and ^18^F-PSMA-1007. EJNMMI Res. 2018;8:30. doi:10.1186/s13550-018-0382-8.

3. Iovkova L, Wangler B, Schirrmacher E, Schirrmacher R, Quandt G, Boening G, et al. para-Functionalized aryl-di-tert-butylfluorosilanes as potential labeling synthons for ^18^F radiopharmaceuticals. Chemistry. 2009;15:2140-7. doi:10.1002/chem.200802266.

4. Robu S, Schottelius M, Eiber M, Maurer T, Gschwend J, Schwaiger M, et al. Preclinical Evaluation and First Patient Application of ^99m^Tc-PSMA-I&S for SPECT Imaging and Radioguided Surgery in Prostate Cancer. J Nucl Med. 2017;58:235-42. doi:10.2967/jnumed.116.178939.
